# Dauer life stage of *Caenorhabditis elegans* induces elevated levels of defense against the parasite *Serratia marcescens*

**DOI:** 10.1038/s41598-019-47969-w

**Published:** 2019-08-09

**Authors:** P. Signe White, McKenna J. Penley, Aimee R. Paulk Tierney, Deanna M. Soper, Levi T. Morran

**Affiliations:** 1 Population Biology, Ecology, and Evolution Graduate Program, Emory University, Atlanta, GA 30322 USA; 2 Department of Biology, Emory University, Atlanta, GA 30322 USA; 3 Microbiology and Molecular Genetics Graduate Program, Emory University, Atlanta, GA 30322 USA; 4 Biology Department, University of Dallas, Irving, TX 75062 USA

**Keywords:** Evolutionary developmental biology, Evolutionary ecology

## Abstract

Host-parasite research often focuses on a single host life stage, yet different life stages may exhibit different defenses. The nematode *Caenorhabditis elegans* has an alternate dispersal life stage, dauer. Despite dauer’s importance in nature, we know little of how it responds to parasites. Previous research indicates that non-dauer *C*. *elegans* prefer to consume the virulent bacterial parasite, *Serratia marcescens*, when given a choice between the parasite and benign *Escherichia coli*. Here, we compared the preferences of dauer individuals from six strains of *C*. *elegans* to the preferences of other life stages. We found that dauer individuals exhibited reduced preference for *S*. *marcescens*, and dauers from some strains preferred *E*. *coli* to *S*. *marcescens*. In addition to testing food preference, a mechanism of parasite avoidance, we also measured host mortality rates after direct parasite exposure to determine if life stage also altered host survival. Overall, dauer individuals exhibited reduced mortality rates. However, dauer versus non-dauer larvae mortality rates also varied significantly by host strain. Collectively, we found evidence of dauer-induced parasite avoidance and reduced mortality in the presence of a parasite, but these effects were strain-specific. These results demonstrate the importance of host life stage and genotype when assessing infection dynamics.

## Introduction

Parasites and hosts impose strong selection on one another. Infections in nature are nearly ubiquitous and host defense against infection is critical in order to survive and reproduce. Hosts primarily use avoidance, resistance, and tolerance as different means of defense. While researchers typically treat each as a separate response, all exist in an interconnected web that acts to prevent or mitigate parasite infection. Avoidance is typically defined as the detection of a parasite and a subsequent behavioral response to either move from an area where a parasite is located or temporarily pause ingestion of resources. Avoidance is often employed as a first line of defense, relative to either resistance or tolerance.

When a host retreats from an area with parasites, it can minimize the risk of infection. Hosts detect and avoid parasites in a variety of ways, including olfactory sensing of parasite toxins, altering food choice from those with potential toxins or high ${{\rm{CO}}}_{2}$ levels, and avoiding areas previously known to harbor infection^1–3^. Some avoidance behaviors may be dependent on the life stage of the host, and thus a host’s chances of survival may differ throughout the host’s life^4,5^. For example, very young organisms may not be able to travel far due to small size or lack of mature phenotype to transport them from harm (e.g. flying)^6^. Importantly, when employed successfully, many of these defense strategies prevent the host from becoming infected, so potentially costly immune responses are not always necessary to stave off infection.

Parasites infect hosts at multiple host life stages with varying effects, yet it is common for researchers to focus on a single host life stage when assessing infection dynamics. There are many examples of parasite infections varying in severity between different host life stages. For example, Balla *et al*. found that when larvae of the host *Caenorhabditis elegans* were infected with an intracellular microsporidia parasite at the first larval stage (L1), the larvae exhibited significantly reduced fecundity as compared with hosts that were infected at the fourth larval stage (L4)^7^. The larvae of the Indian meal moth *Plodia interpunctella*, a commonly studied host organism in host-parasite interactions, suffers high mortality rates when infected by a granulosis virus, whereas adults do not appear to be infected at all^8^. Alternatively, some diseases only affect adults, such as in the high mortality of adult female cassava mites (*Mononychelles tanajoa*) infected with a fungal pathogen, *Neozygites floridana*, which leaves other life stages unharmed^9^. Host life stage may be an important variable when analyzing infection dynamics and host defense. In this study, we aimed to determine whether exposure to parasites at different host life stages may result in varying responses in host defense strategies in the nematode, *C*. *elegans*.

*C*. *elegans* is a model host organism that has been used to understand innate immunity and infection^10–12^. In the wild, *C*. *elegans* live on a variety of decomposing organic materials, and thus come into contact with many types of microorganisms, whether pathogenic, beneficial, and benign^11,13^. Because of the variety of microbes present in the worm’s natural environment, *C*. *elegans* must be able to discriminate between food sources and potential pathogens or potentially incur reductions in fitness. Many different bacterial parasites are capable of infecting *C*. *elegans*^14–17^. In previous studies, *C*. *elegans* have shown a surprising attraction to the pathogenic bacteria *Serratia marcescens* relative to the benign food source, *E*. *coli* OP50^18,19^. *S*. *marcescens* have been found in the same sampled natural substrates as *C*. *elegans* populations and thus is likely to be consumed by *C*. *elegans* in some environments^11,19^. It is possible that *C*. *elegans* has an evolutionary history with *S*. *marcescens*^13^. Further, it is also possible that isolated *C*. *elegans* populations have adapted to their local parasites (or vice versa), whereby we see variation in response to *S*. *marcescens* due to host or parasite genotypes^20,21^. The strain of *S*. *marcescens* used in Pradel *et al*. (2007)^19^ and Zhang *et al*. (2005)^18^ is a virulent parasite, which can significantly shorten the average life span of infected N2 L4 worms^22^. It is likely that initial attraction to *S*. *marcescens* is elicited by the molecule lipodepsipentapeptide serrawettin W2, which is required for spreading growth in certain strains of *S*. *marcescens*^19^. Despite this initial attraction to *S*. *marcescens*, *C*. *elegans* is able to exhibit learned avoidance behavior of the parasite after approximately 4 hours of exposure^12,18^. Nonetheless, several hours of feeding on a parasitic food source may be sufficient for infection^23^. It would seem that innate repulsion from *S*. *marcescens* could be a more advantageous defense strategy than learned avoidance.

Previous assessments of *C*. *elegans* bacterial preference have been performed on adults and L4 larvae, which may experience weak selection for bacterial preference because these life stages are not known to be associated with dispersal in nature. Interestingly, the vast majority of natural samples of *C*. *elegans* have been isolated in dauer, a stage of developmental arrest induced by resource scarcity and crowding^11,24^. The dauer life stage takes the place of a molting stage between larval stage 3 (L3) and larval stage 4 (L4)^25^. Importantly, the dauer life stage is non-feeding, and thus relies on chemotaxis to discern environmental cues. Previous work has shown that dauer individuals have a greater attraction towards CO_2_ than do adult individuals, which may signal to the dauer worms a nearby bacterial food source^26^. Conversely, a study by Albert and Riddle (1983) found that dauer worms are unresponsive to sodium ions (Na+) compared with non-dauer life stages^27^. Thus, multiple sensory avenues have been found to differ between various *C*. *elegans* life stages.

Physiological processes which induce development into dauer are elicited by pheromones and environmental cues, particularly crowding and food limitation^28^. Dauer individuals can survive exposure to starvation, heat, and desiccation stress for much longer periods of time than all other life stages of *C*. *elegans*^28^. Dauer is phenotypically unique compared to other larval stages, and dauer induction is known to induce specific changes in gene expression throughout the genome relative to the other *C*. *elegans* life stages^29^. In nature, the dauer life stage is thought to facilitate the colonization of new food sources and thus selection may favor dauer individuals that can successfully discriminate between benign and pathogenic bacteria^13^. Therefore, parasite avoidance could be a function of the dauer life stage, while other life stages may exhibit more indiscriminate feeding.

Here, we tested the effects of *C*. *elegans* life stage on food preference during interactions between *C*. *elegans* hosts and the bacterial parasite, *Serratia marcescens*. Specifically, we were interested in the effects of *C*. *elegans* dauer stage, relative to other host life stages, on its ability to discriminate between benign and pathogenic food sources. We assayed food preference in the hosts by comparing behavioral responses of each host life stage to a choice between pathogenic *S*. *marcescens* (Sm2170) and benign *Escherichia coli* (OP50). We next compared individuals, including L4 and adult worms, which had emerged from dauer and resumed development as compared with those that did not transition to dauer during development. Then, we assessed the effects of direct exposure to Sm2170 on dauer mortality as a means of measuring the effects of host life stage on host defense overall. All of these experiments were performed in CB4856 and N2, both of which are lab-adapted strains and the subject of previous studies on *C*. *elegans*-pathogen interactions. Additionally, we utilized 4 natural isolates, all of which had been collected in dauer and reared in the lab for fewer than 15 generations, to determine the effects of dauer on host defense within isolates from different types of substrates and geographic areas.

## Methods

### Bacterial strains

The primary food source of *C*. *elegans*, *Escherichia coli* OP50, was acquired from the Caenorhabditis Genomics Center (CGC, University of Minnesota) in 2010 by L.T.M, frozen at −80 °C, and periodically thawed for each new experiment. The strain of *S*. *marcescens* used, Sm2170, was acquired from Curt Lively at Indiana University in 2010, also frozen at −80 °C, and then thawed for each new experiment. The experimental strain of *Escherichia coli* HB101 was acquired from Steve L’Hernault at Emory University in 2018.

### *C. elegans* strains

N2 and CB4856 are both common laboratory strains of *C*. *elegans* that L.T.M originally received from the CGC in 2010. The four natural strains used in our experiments were all isolated in the dauer life stage and collected by either Marie-Anne Felix or Matt Rockman and then immediately frozen at −80 °C for later use. JU543 was isolated in 2004 by M.A. Felix from a woodlouse, *Oniscus asellus*, in a rural garden in Primel Tregastel, France. JU2140 was isolated in 2011 by M.A. Felix on a slug in rotting acorns in a forest in La Blanc, France. JU2816 was isolated in 2014 by M.A. Felix on vertebrate feces containing plum remains in an orchard in Orsay, France. QX1233 was isolated in 2007 by M. Rockman from a compost heap in an urban garden in Berkeley, CA.

### Choice assays

The dauer life stage replaces the L3 life stage of non-starved individual worms, thus most of our comparisons are between L3 and dauer individuals. Since previous work has focused mostly on the preference of adult individuals, we also rely heavily on comparisons between adult and dauer.

Choice assays were modeled after Zhang *et al*.^18^ and Glater *et al*.^30^. For each worm strain gravid adults were bleached using standard laboratory bleach and NaOH methods to isolate eggs^31^. Half of the eggs were then placed on a 10 cm NGM-Lite plate seeded with *Escherichia coli* OP50, then placed in a 20 °C incubator and allowed to grow to the desired developmental stage^32^. The other half of the eggs were also placed on a 10 cm OP50-seeded NGM-Lite at 20 °C and allowed to go into dauer arrest. After 2 weeks of starvation at 20 °C, nearly all surviving individuals developed into dauer. This process repeated for each *C*. *elegans* strain. Using a time to maturity chart and visual cues, we waited the amount of time it takes on average for eggs to hatch then go through each subsequent developmental stage^33^. We visually checked to see if a substantial majority of individuals (>85%) were in the appropriate life stage at the corresponding time. The L3 larvae have not yet formed a vulva, which differentiates them from L4. Dauer individuals have a unique phenotype permitting easy identification. No worms had prior exposure to Sm2170. The choice assay plate was the first encounter the nematodes had with *S*. *marcescens*. Finally, to acquire post-dauer L4 and post-dauer adult individuals, we placed dauer naïve to *S*. *marcescens* on an *E*. *coli* OP50 plate to resume development (development into L4 resumes approximately 16 hours after feeding^25^, and development into adult occurs after 36 hours). To prepare the bacteria for the choice assay, *E*. *coli* OP50 and *S*. *marcescens* 2170 were grown overnight in liquid LB at 28 °C. Overnight growth in OP50 and Sm2170 results in a mean OD600 of 1.5 (55 × 10^8^ CFUs) for OP50 and a mean OD600 of 1.0 (7.8 × 10^8^ CFUs) for Sm2170. 25 μl of each bacteria was placed on the opposite sides of an unseeded 10 cm NGM-Lite plate (Supplementary Figure 1). For each treatment, the worms were washed 3x in M9 buffer to remove external OP50, then approximately 100 worms were placed directly in the center of each plate. After two hours, the worms were counted under a dissecting microscope to determine how many individuals were on Sm2170, OP50, or elsewhere on the plate. The recorded counts were used to calculate the choice index (CI):

$$\frac{\#\,on\,Sm2170-\#\,on\,OP50}{\#\,total\,number\,plated}$$

Previous choice indices have used the total number of worms that chose one bacterium or the other, excluding or including those that did not choose^18,30^. Glater *et al*. found <5% of the worms did not make a decision for either bacteria, thus excluding them from analysis. However, because we found >5% make neither decision, we included all individuals plated in our calculations (Supplementary Figure S1).

This was repeated for all 6 strains and for 6 life stages: L3, dauer, L4, post-dauer L4, adult, and post-dauer adult – for a total of 36 treatments. Each treatment was repeated 10 times for a total of 360 replicates. The same choice assay methodology was repeated for assessing preference of N2 and CB4856 between *Escherichia coli* HB101 vs OP50. In this assay, the same number of worms was used to analyze preference as in our previous assays: 100 individuals of either worm genotype and either life stage (L3 or dauer), for a total of 4 treatments with 10 replicates (40 replicates total).

### Statistical analysis

**Figure 1:**
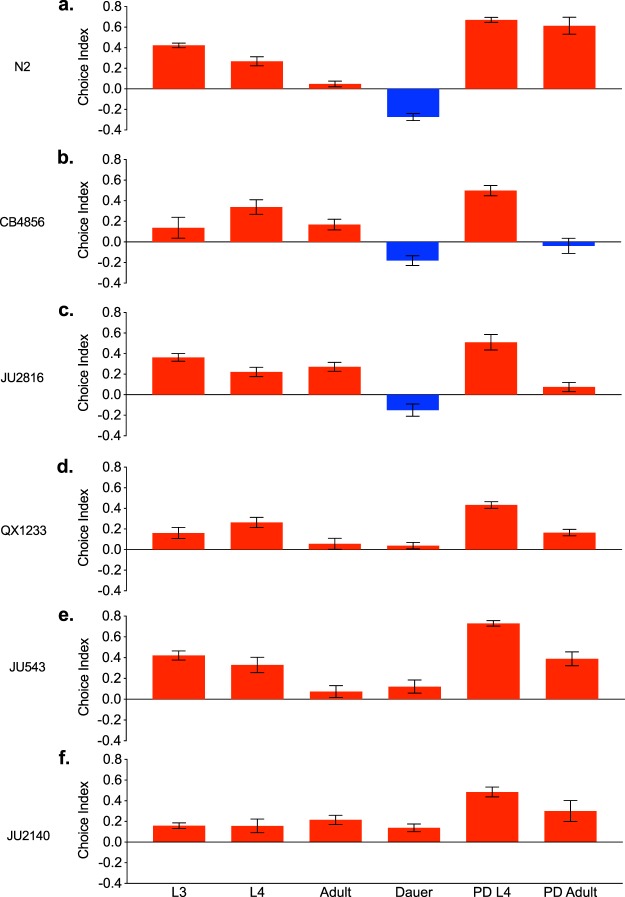
(**a–f**) Sm2170 and OP50 choice assays in all strains and life stages. CI < 0 (blue) are found in higher proportions on OP50, CI > 0 (red) are found in higher proportions on Sm2170, whereas a score of CI = 0 indicates no preference. Each bar represents an average of 1000 worms (100 worms per plate x 10 replicates). The vertical lines within each bar depict the standard error of the mean (SEM). PD = post dauer.

JMP Pro13 (SAS Institute, Cary, North Carolina) was used to perform statistical analyses on the data. The bacterial choice data were transformed into binomial data (yes = chose Sm2170, no = chose either OP50 or nothing) using the OFFSET function in Excel (Microsoft, Redmond, Washington). In addition, we transformed binomial data on whether or not worms chose any bacteria (yes = chose Sm2170 or OP50, no = chose neither) to determine if there were differences between strains and life stages in whether or not any choice was being made. In both analyses, we used binomial data as opposed to performing analyses on ratio means. Using this binomial data, we then used JMP Pro13 to perform a success/failure generalized linear model (GLM) with a binomial distribution and logit link function (strain, life stage, and strain by life stage effects on bacterial choice). We tested for overdispersion using the Pearson chi-squared test but did not detect significant overdispersion. Contrast tests were run between each strain, life stage, strain by life stage, and replicate population effects for both binomial datasets. We present the data as choice indices below (Fig. 1) and as the proportion that chose either or neither bacteria (Supplementary Figure 1). Standard linear models may be inappropriate in the case of our choice index data as they are discrete data with upper and lower bounds of 1 and −1. However, because many of our response variable values are not close to the bounds, an ANOVA can still perform well. Thus, we ran a two-way ANOVA in JMP Pro13 for all CI data and performed Tukey’s multiple comparisons tests. The GLM and the ANOVA models yielded results that were qualitatively in agreement. Here, we present the ANOVA results because our figures present CI data. In addition, we ran an ANOVA for preference between the benign strains of *Escherichia coli* HB101 and OP50 in L3 and dauer worms. The nematodes had been raised for many generations on the OP50 strain, so we tested to see if preference was due to familiarity with the OP50 strain by comparing their attraction to it with the HB101 strain. These data were also analyzed in JMP Pro13 as described above.

### Mortality assays

In addition to behavioral avoidance, we wanted to test whether dauer was better able than non-dauer L3 individuals at surviving *Serratia marcescens*. In other words, does an increase in avoidance of the parasite also confer an increase in resistance? In these assays, we grew a lawn of *S*. *marcescens* from each treatment, one treatment per plate, and then allowed worms to become infected by placing them on the treatment bacteria. By placing the worms directly on the *S*. *marcescens* lawn, but maintaining the possibility of escape from the lawn, we allow for survival via avoidance mechanisms. Further, individuals that ingest the parasite have the opportunity to also resist or tolerate infection. Mortality assays were performed using all abovementioned *C*. *elegans* strains but only in the dauer and L3 life stages. To prepare the assay, one-third of a 10 cm petri dish containing NGM-Lite (US Biological, Salem, Massachusetts) was seeded with 35μl OP50 grown up overnight in liquid LB at 28 °C. The opposite one-third was seeded with 35μl of Sm2170 or heat-killed Sm2170 (the control) as described in Penley and Morran^34^. The middle one-third was kept unseeded. These plates were then grown up in 28 °C overnight. Before worms were plated, 20μl of 100 mg/ml Ampicillin antibiotic was placed in the middle of the plate to prevent the spread of Sm2170. To achieve the desired life stage, egg hatches were synchronized as done for the choice assays above. Worms at the desired life stage were washed 3x with M9 buffer to remove external OP50, counted, then placed directly on the Sm2170 (or heat-killed Sm2170). Plates were then incubated for 48 hours at 20 °C. Subsequently, numbers of living worms were then counted by assessing movement and prodding with a platinum wire^35–38^. We then repeated this procedure with heat-killed Sm2170 as a control. Mortality assays directly assess the host’s ability to defend against Sm2170 infection by capturing a total measure of host defense, whether by avoidance, resistance and/or tolerance. These mortality assays were repeated 10 times for all six strains in both L3 and dauer individuals, for a total of 600 individuals tested for mortality per strain per life stage.

We calculated mortality rates as the proportion of hosts dead on the treatment plates out of the average total number alive on the control plates to account for any host mortality that did not occur due to the parasite.

### Statistical analysis

JMP Pro13 was again used to perform statistical analyses on the data. Nonparametric Wilcoxon tests were performed to determine significance between all treatment groups (each strain: CB4856, N2, JU543, JU2816, JU2140, JU543, and life stage: dauer and L3), and one-way chi square values are reported. Similar to the choice indices, the parameter in this model is a single outcome, whether the worms either died (=yes) or survived (=no). Here, we corrected for multiple statistical tests (two tests: either treatment group or life stage) using a Bonferroni-corrected p-value of 0.025 (0.05/2).

## Results

### Choice indices (CI)

We tested the effect of *C*. *elegans* life stage on bacterial food preference using the relatively benign food source, *E*. *coli* OP50, and the virulent parasite, *S*. *marcescens* Sm2170. Food preference was quantitatively measured using a choice index (CI). A mean positive CI indicates a preference for the parasite, whereas a mean negative CI indicates a preference for the benign food source. A CI nearing zero indicates there is no preference for either.

### Life stage effects

By comparing *C*. *elegans* dauer individuals with L3 individuals across all six strains, we found a significant difference in preference between the two life stages (*F*_*1*,*502*_ = 76.0173, p < 0.0001) (Table S1). Specifically, L3 individuals exhibited a strong preference for *S*. *marcescens* Sm2170 (CI = 0.278). However, dauer individuals exhibited a very slight mean preference for *E*. *coli* OP50, albeit essentially a lack of preference between the bacteria (CI = −0.008). Nonetheless, dauer individuals exhibited a significant decrease in their attraction to the parasite relative to L3 individuals (Fig. 1, Table S2).

In addition, when comparing dauer individuals with adult individuals across all strains, dauers exhibited significantly reduced preference for Sm2170 relative to adults (*F*_*1*,*502*_ = 34.4982, p < 0.0001) (Table S1). Adults had an overall CI = 0.133 compared with the aforementioned CI = −0.008 for dauers (Table S2). Overall, we found a greater mean CI value for all other life stages (including L4, post-dauer L4, and post-dauer adult) compared with dauer (Table S2), suggesting that dauer is unique. Further, we calculated the proportion of individuals that made a choice (chose either bacterium) versus those that made no choice (remained on the unseeded areas of the plate). Across all strains, only 18% of dauer worms did not choose a bacterium, whereas 44.3% of L3 individuals did not choose (p < 0.0001). Dauer chose one or the other bacterium at a much higher rate than did all the non-dauer life stages (Fig. S1; p < 0.0001). Thus, the lack of a strong preference exhibited by dauer individuals is not the result of few individuals making a choice.

### *C*. *elegans* strain effects

**Figure 2:**
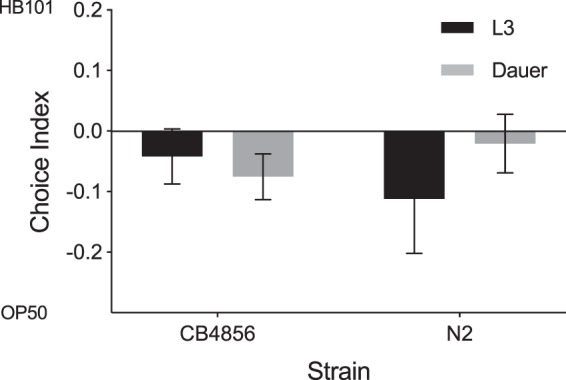
Choice assays between OP50 and another benign *E*. *coli* strain, HB101 tested with *C*. *elegans* strains CB4856 and N2 in the L3 and dauer life stages. A two-way ANOVA found no significant difference across either strain ($p=0.8989$), life stage ($p=0.6278$), or the interaction between the two ($p=0.6278$) in preference for OP50 over HB101. Error bars represent SEM.

In addition to comparisons across life stages, we also assessed whether different strains of *C*. *elegans* differed in their preference for *S*. *marcescens* Sm2170 and *E*. *coli* OP50. We found significant effects of worm strain on bacterial choice (F_1,502_ = 146.3386, p < 0.0001) (Table S1). Averaged across all life stages, CB4856 had the lowest CI, while JU543 had the highest CI (Figs 1b and 2e; Table S2). Additionally, we observed significant strain by life stage effects (F_1,25_ = 4.4523, p < 0.0001) (Table S1 and S3). The dauer life stage of the N2, CB4856, and JU2816 strains showed an overall preference for *E*. *coli* (CIs = −0.274, −0.181, and −0.015, respectively), while the other strains (QX1233, JU543, and JU2140) had a preference for *S*. *marcescens* Sm2170 while in dauer (CIs = 0.038, 0.122, and 0.138, respectively) (Fig. 1a–f, Table S2).

### OP50 vs. HB101

One potential caveat to our results is that rather than an aversion to Sm2170, we may have detected an attraction of dauer toward OP50, particularly in our N2 and CB4856 strains. Such attraction could be a result of lab adaptation, as OP50 serves as the standard laboratory food source and N2 and CB4856 have been maintained on OP50 for many generations. To distinguish between Sm2170 avoidance and OP50 preference, we exposed CB4856 and N2 dauer worms to both *E*. *coli* OP50 and *E*. *coli* HB101, a similarly benign strain of bacteria. We compared the choices of dauer individuals with that of L3 individuals in both CB4856 and N2 strains to determine if dauers consistently preferred OP50. We found no significant difference in preference between life stages or strains (F_1,35_ = 1.906, p = 0.1762, power = 75.2% to detect a CI difference of 0.1), nor any strong preference for OP50 (Fig. 2). Thus, the CI differences exhibited by N2 and CB4856 dauer individuals in the presence of *S*. *marcescens* (Fig. 1), relative to other life stages, are driven by parasite avoidance.

### Mortality assays

**Figure 3:**
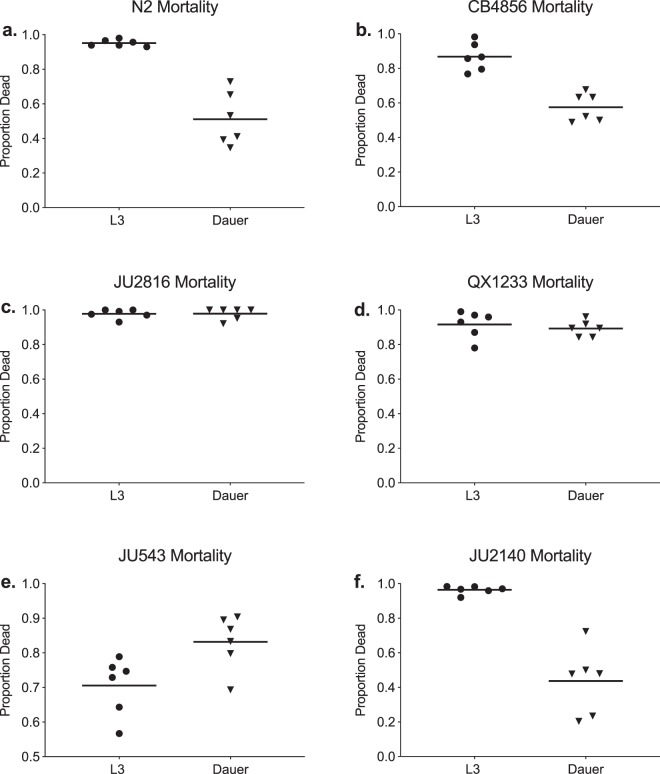
(**a–f**) Mortality of L3 and dauer individuals 48 hours after placement of individuals directly on Sm2170. Horizontal bar shows the mean proportion dead across all replicates. Significant differences in survival between dauer and L3 while on the pathogen were observed for (**a**) N2 $({X}_{1}^{2}=8.3368,\,p=0.0039)$, (**b**) CB4856 (${X}_{1}^{2}=8.3368,\,p=0.0038)$, and (**f**) JU2140 (${X}_{1}^{2}=7.4103,\,p=0.0065$).

While we observed diminished attraction to Sm2170 in most dauer individuals across strains, we next determined if dauer individuals were better able to survive when directly exposed to Sm2170. To test this, we performed mortality assays on both L3 and dauer individuals and compared mortality rates across all *C*. *elegans* strains. We found that dauer individuals exhibited significantly lower mean mortality rates when exposed to *S*. *marcescens* Sm2170 than did L3 individuals when averaged across all strains (${X}_{1}^{2}=9.1513,\,p=0.0025$). There were also significant effects of strain on mortality rates (${X}_{5}^{2}=34.1737,\,p=0.0001$). N2 and CB4856 both exhibited reduced mortality in dauer (N2 had a 0.255 reduction in mortality when in dauer, CB4856 had a 0.334 reduction); whereas the natural isolates varied in their responses (Fig. 3, Table S5).

## Discussion

It is possible to miss important insights about an organism if its life history and ecology are not taken into consideration in laboratory experiments, particularly in the case of novel host-parasite interactions. Despite *C*. *elegans* fundamental use as a model to advance the biological sciences, the ecology of *C*. *elegans* has rarely been taken into account when designing and interpreting experiments. Our work aims to highlight the importance of incorporating observations from nature and applying more ecological perspective to future host-parasite research. Here, we show the life stage of a host upon encountering a parasite may determine both the host’s mechanism of defense and the host’s overall level of defense. Previous studies have found that late larval (L4) and early adult *C*. *elegans* are preferentially attracted to the parasite *S*. *marcescens* when given a choice between the parasite and their benign food source, *E*. *coli*^18,30^. Some have posited that this attraction may be due to behavioral manipulation of *C*. *elegans* by *S*. *marcescens* as a type of “trojan horse” mechanism known to occur in other bacterial pathogens^39^. However, these studies did not assess individuals in the alternative dauer life stage, which is critical for dispersal in *C*. *elegans*^11^. We hypothesized that dauer individuals would exhibit a decreased preference for the parasite, *S*. *marcescens*, and increased preference for the benign food source, *E*. *coli*, due to dauer’s role in the colonization of new habitats. Here, we found that dauer individuals overall exhibited significantly reduced preference for *S*. *marcescens* relative to other life stages of *C*. *elegans* (Fig. 1). Further, several strains exhibited a mean preference for *E*. *coli* relative to *S*. *marcescens*, whereas other strains showed no preference (a CI close to 0), or still slightly preferred *S*. *marcescens*. (Fig. 2). Importantly, increased dauer preference for *E*. *coli*, relative to L3 individuals, was conditional on the presence of *S*. *marcescens* (Figs 1 and 2), therefore the altered food preference exhibited by dauer individuals functions as a form of parasite avoidance. Our results are also consistent with Glater *et al*.^30^, in which CB4856 adults were found to have a low CI. N2 and CB4856 had an overall lower preference for *S*. *marcescens* Sm2170 compared with natural isolates (lab strains had an average CI = 0.181, natural isolates had an average CI = 0.245) (Table S2). We then addressed whether dauer individuals exhibited greater levels of host defense, relative to L3 larvae. We found that, overall, the mean mortality rates exhibited by dauer individuals were reduced. However, this effect of dauer was largely strain specific. Thus, *C*. *elegans* life stage can alter the mechanism of defense employed by the host via parasite avoidance. Further, dauer individuals can also exhibit increased levels of host defense.

We observed both increased parasite avoidance at the dauer life stage, as well as increased levels of host defense. Did parasite avoidance reduce levels of host mortality? The most substantial decreases in preference between L3 and dauer individuals were in both of the laboratory-adapted strains, CB4856 and N2 (Fig. 1a,b). Interestingly, both CB4856 and N2 also exhibited greater levels of host defense (Fig. 3a,b). However, JU2816, had a significantly increased preference for OP50 when in dauer (Fig. 1c), but both L3 and dauer individuals died at high rates when directly exposed to the parasite (Fig. 3c). Conversely, strain JU2140 exhibited high levels of host defense (Fig. 3f) without parasite avoidance (Fig. 1f). Importantly, the mortality assays were constructed to permit parasite avoidance after initial exposure. However, the nematodes were directly exposed to the parasite to begin the assay, therefore the hosts cannot avoid the parasite altogether which may reduce the efficacy of avoidance. Regardless, taken together, these results suggest that dauer can impact both avoidance and overall defense. Specifically, avoidance may contribute to the substantially greater levels of host defense in CB4856 and N2 dauer individuals by facilitating avoidance of the parasite. However, increased host defense in the dauer stage is unlikely to be solely driven by avoidance because the JU2140 strain exhibits increased defense in the absence of avoidance.

In nature, dauer individuals likely discriminate between benign and pathogenic food sources. Therefore, parasite avoidance behavior at the dauer stage may be under strong selection. Although L4 larvae generally exhibit a preference for *S*. *marcescens*, previous work has shown that L4 individuals can evolve elevated levels of parasite avoidance when experimentally evolved in the presence of *S*. *marcescens*^34^. Thus, L4 individuals do not necessarily lack the ability to detect a parasite, but it is plausible that selection primarily acts on the dauer stage rather than the L4 stage in nature. Importantly, post-dauer L4’s and adults generally do not exhibit parasite avoidance (Fig. 1, but see Fig. 1b), indicating that dauer induction is not sufficient for parasite avoidance compared with worms that develop normally (i.e. go through the L3 life stage instead). Curiously, post-dauer L4 individuals, in particular, have a much greater preference for the parasite than do dauer individuals (Figs 1 and S1). Clearly, food preference is substantially different in the dauer life stage, but only transiently, and it appears that avoidance of Sm2170 is constrained to the dauer stage. Yet, our data also indicate that dauer induction may alter food choice in subsequent life stages.

An important caveat to note about our experimental design is the way in which we induced dauer development. As opposed to many previous dauer studies that induced dauer formation via chemical cues^40–43^, here we allowed populations to exhaust their lawn of *E*. *coli*, experience overcrowding, and starve for a period of two weeks. Importantly, *C*. *elegans* maternal effects can induce parasite avoidance via the induction of diapause in the offspring of mothers that were exposed to the parasite^44^. It is plausible that starved mothers may produce dauer offspring with greater levels of parasite avoidance relative to dauer individuals that were induced chemically. Therefore, our results may depend on dauer induction via overcrowding and starvation. Nonetheless, dauer induction via crowding and starvation is likely more relevant to the boom-and-bust conditions *C*. *elegans* encounter in nature than exposure to concentrated dauer pheromone^11^.

While our data supports dauer individuals’ ability to discriminate between a benign versus a harmful bacterium, it may be the case that local genotype by genotype (GxG) or genotype by environment (GxE) interactions are responsible for the variation between strains in both parasite avoidance and defense^21^. *C*. *elegans* in nature may be locally adapted to its parasites, and thus recognize local parasites more effectively than foreign parasites^45^. While it is plausible that natural isolates of *C*. *elegans* encounter *S*. *marcescens* and other *Serratia* species in nature, they likely have not encountered the Sm2170 strain used in this study^13^. Rather, some host strains may have responded to serrawettin W2 produced by Sm2170 more than other host strains due to their specific evolutionary histories with parasites. Conversely, the strain differences we observed may largely be due to genetic drift, rather than signatures of local adaptation. To assess the contributions of selection versus drift, it would be ideal to compare the substrates in which the *C*. *elegans* isolates were collected to determine the natural variation between strains of bacterial parasites and their associated *C*. *elegans* hosts. Nonetheless, our results show the importance of extending analysis beyond the standard lab strains to incorporate natural isolates, without which we would not have observed substantial variation in response to the parasite.

This study demonstrates the importance of considering both the natural history and ecology of *C*. *elegans* as well as its facultative life stage in understanding laboratory studies of host-parasite interaction dynamics. Without testing dauer, researchers would continue to assume an overall attraction of *C*. *elegans* to *S*. *marcescens*. This work provides a step in better elucidating the unique differences of *C*. *elegans* dauer life stage and the role that *C*. *elegans* life history may play in nature. Future work could assess the effects of dauer on long-term population growth and the evolution of host defense by passaging both wildtype and dauer-deficient mutant strains in the presence of parasites. In addition, while our work focused solely on *S*. *marcescens*, there is evidence to suggest that many types of parasites naturally infect worm populations. Therefore, it is critical to determine if parasite avoidance is a general response in *C*. *elegans* dauers, or if avoidance is highly specialized. Furthermore, how do olfactory responses to parasites change throughout a worm’s lifetime, and what ramifications do these various responses have in natural populations?

Beyond *C*. *elegans*, each life stage of a particular host may hold differing strategies of ridding itself of parasites or avoiding them altogether. Particular life stages may play specific roles in nature^7–9^ that could shape the evolution of defense mechanisms that they employ. When only a single host life stage is measured, important dynamics of the host-parasite interaction may be missed. Our work shows how a developmental life stage that is essential in nature, but not in the lab, is able to avoid parasites and contribute substantially to host defense. The effects of life stage may be more common than we know in a wide array of species and may be worth examining more rigorously. This approach may be of particular importance to species with dispersal life stages in which fitness strongly depends upon avoiding infections in newly colonized habitats.

## Supplementary information

Supplementary Tables 1-5 and Supplementary Figure 1

## Data Availability

We will submit our raw data files to Dryad upon acceptance of this manuscript for publication.
