# Supplementary material for: Dauer life stage of Caenorhabditis elegans induces elevated levels of defense against the parasite Serratia marcescens
Source: Sci Rep. 2019 Aug 9;9:11575. doi: 10.1038/s41598-019-47969-w (PMC6688991; doi:10.1038/s41598-019-47969-w)
Supplement: Supplementary file 1 — Supplementary Tables 1-5 and Supplementary Figure 1 [file 41598_2019_47969_MOESM1_ESM.pdf]

Supplementary Information

Dauer life stage of *Caenorhabditis elegans* induces elevated levels of defense against the parasite *Serratia marcescens*

P. Signe White*, †, McKenna J. Penley †, Aimee Paulk Tierney ‡, Deanna M. Soper § & Levi T. Morran †

**Population Biology, Ecology, and Evolution Graduate Program, Emory University, Atlanta, GA 30322, USA.*
†Department of Biology, Emory University, Atlanta, GA 30322, USA.
‡Microbiology and Molecular Genetics Graduate Program, Emory University, Atlanta, GA 30322, USA
§Biology Department, University of Dallas, Irving, TX 75062, USA.

Supplementary Figure S1

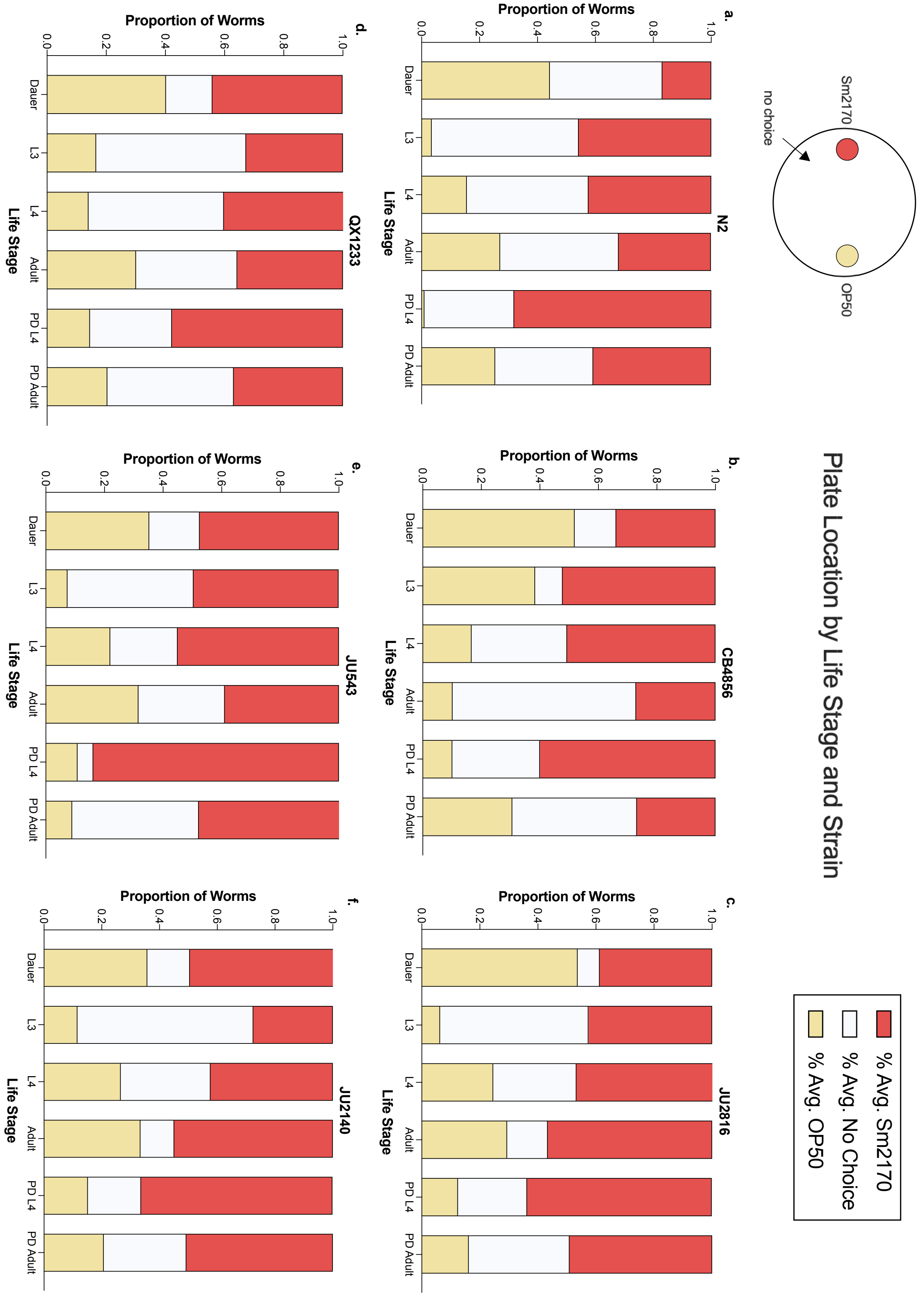

Supplementary Table S1
Choice Assay Data – ANOVA

Whole Model
Two-way ANOVA,
standard least-squares Alpha: 0.05

Analysis of Variance

**Source**	**DF**	**SS**	**Mean Square**	**F Ratio**	
Model	35	20.658067	0.590230	12.7114	
Error	502	23.309356	0.045433	**Prob > F**	
C. Total	537	43.967423		<0.0001	
**Effect Tests**	**Nparm**	**DF**	**SS**	**F Ratio**	**P value**
Interaction	25	25	5.168361	4.4523	P<0.0001
Life Stage	5	5	15.512685	66.8175	P<0.0001
Strain	5	5	1.766846	7.6103	P<0.0001

Contrast Tests: Tukey's Multiple Comparisons

**Life Stage**	**SS**	**NumDF**	**DenDF**	**F Ratio**	**Prob > F**
Dauer vs. L3	3.53	1	502	76.0173	<0.0001
Dauer vs. Adult	1.602	1	502	34.4982	<0.0001
Dauer vs. all other life stages	6.795	1	502	146.3368	<0.0001

Supplementary Table S2
Choice Indices

Strain	L3	Dauer	L4	PD L4	Adult	PD Adult	Averages
JU543	0.421	0.122	0.330	0.730	0.074	0.389	**0.284**
JU2816	0.363	-0.150	0.222	0.511	0.272	0.099	**0.241**
QX1233	0.161	0.038	0.263	0.433	0.056	0.165	**0.207**
JU2140	0.159	0.138	0.157	0.485	0.215	0.301	**0.248**
CB4856	0.138	-0.181	0.339	0.499	0.169	-0.038	**0.171**
N2	0.423	-0.274	0.268	0.671	0.048	0.154	**0.190**
**Averages**	**0.278**	**-0.008**	**0.263**	**0.529**	**0.133**	**0.191**	**0.227**

Dark orange denotes natural isolates. Green text denotes lab strains.

Supplementary Table S3
Choice Assay Data – GLM Binomial

Life Stages: L3, L4, Adult, Dauer, PD L4, and PD Adult
Strains: CB4856, N2, JU543, JU2816, JU2140, QX1233

Whole Model Test

**Model**	**-Log Likelihood**	**L-R ChiSquare**	**DF**	**Prob > ChiSq**
Difference	2414.9995	4829.999	60	<0.0001*
Full	33136.7724			
Reduced	35551.7719			
**Goodness of Fit Statistic**	**ChiSquare**	**DF**	**Prob > ChiSq**	
Pearson	52359.85	51863	0.0618	
Deviance	66273.54	51863	<0.0001*	

Effect Tests

**Source**	**DF**	**L-R ChiSquare**	**Prob > ChiSq**
Strain	5	439.7864	<0.0001*
Life Stage	5	2280.6587	<0.0001*
Replicate	25	789.01358	<0.0001*
Strain*Life Stage	25	1814.0432	<0.0001*

Contrast Test All Strains: Dauer vs. Non-Dauer

**Life Stage Overall**	**-Log Likelihood**	**DF**	**L-R ChiSquare**	**Prob > ChiSq**
L3 vs. Dauer	33493.604713	1	713.66463197	3.1936e-157

Supplementary Table S4

Choice Assay Data (chose either bacteria or chose neither) – GLM Binomial

Life Stages: L3, L4, Adult, Dauer, PD L4, and PD Adult
Strains: CB4856, N2, JU543, JU2816, JU2140, QX1233

Whole Model Test

Model	-Log Likelihood	L-R ChiSquare	DF	Prob > ChiSq
Difference	3236.52246	6527.045	35	<0.001*
Full	30586.2972			
Reduced	33849.8196			
**Goodness of Fit Statistic**	**ChiSquare**	**DF**	**Prob > ChiSq**	
Pearson	52354.00	52318	0.4549	
Deviance	61172.59	52318	<0.001*	

Effects Tests

Source	DF	L-R ChiSquare	Prob > ChiSq
Life Stage	5	377.56523	<0.0001*
Strain	5	550.72470	<0.0001*
Life Stage*Strain	25	5554.67900	<0.0001*
Replicate	1	44.472647	<0.0001*

Contrast Tests

Life Stage Overall	-Log Likelihood	DF	L-R ChiSquare	Prob > ChiSq
All Life Stages vs. Dauer	30725.623388	1	278.65239522	1.476607e-62
L3 vs. Dauer	30701.904974	1	275.67378993	6.582637e-62
L4 vs. PD L4	30566.817157	1	5.4980566642	0.019
Adult vs. PD Adult	30573.892661	1	19.649063525	9.3048903e-6

Supplementary Table S5
Mortality Assay Data – GLM Binomial

Life Stages: L3, Dauer
Strains: CB4856, N2, JU543, JU2816, JU2140, QX1233

strain
life stage
replicate [strain * life stage] strain * life stage

Whole Model Test

Model	-Log Likelihood	L-R ChiSquare	DF	Prob > ChiSq
Difference	860.190156	1720.380	71	<0.0001*
Full	2960.76507			
Reduced	3820.95522			
**Goodness of Fit Statistic**	**ChiSquare**	**DF**	**Prob > ChiSq**	
Pearson	6885.000	7265	0.9993	
Deviance	5921.530	7265	1.0000	

Effects Tests

Source	DF	L-R ChiSquare	Prob > ChiSq
Strain	5	404.05623	<0.0001*
Life Stage	1	1.5237e-5	0.9969
Replicate	60	339.59913	<0.0001*
Strain*Life Stage	5	321.34359	<0.0001*

Contrast Tests

Life Stage Overall	-Log Likelihood	DF	L-R ChiSquare	Prob > ChiSq
L3 vs. Dauer	2960.7650764	1	0.000015237	0.9968854994

Legend:

Supplementary Figure S1. Mean proportion of individuals exhibiting each potential outcome during choice assays for each strain. These graphs depict the average proportion of individuals in each life stage that went to either OP50 (yellow bar), Sm2170 (red bar), or did not choose either OP50 or Sm2170 (white bar). “No choice” worms were found on the unseeded parts of the plate. Note that dauer is the first bar of each graph. Across all strains, significantly more dauer worms chose one or the other bacteria than did all other life stages ($p = 1.476607e-62$).
